# Raman Spectroscopy Studies on the Barocaloric Hybrid Perovskite [(CH_3_)_4_N][Cd(N_3_)_3_]

**DOI:** 10.3390/molecules25204754

**Published:** 2020-10-16

**Authors:** Rosivaldo Xavier da Silva, Carlos William de Araujo Paschoal, Clenilton Costa dos Santos, Alberto García-Fernández, Jorge Salgado-Beceiro, María Antonia Señarís-Rodríguez, Manuel Sanchez-Andujar, Ariel Nonato Almeida de Abreu Silva

**Affiliations:** 1 Coordenação de Ciências Naturais, Universidade Federal do Maranhão, Campus VII, São Luís 65400-000, Brazil; rosivaldo.xs@ufma.br; 2 Departamento de Física, Universidade Federal do Ceará, Fortaleza 65455-900, Brazil; paschoal.william@fisica.ufc.br; 3 Departamento de Física, CCET, Universidade Federal do Maranhão, São Luís 65085-580, Brazil; clenilton.cs@ufma.br; 4 Departamento de Química, Facultade de Ciencias y CICA, Universidade da Coruña, 15071 A Coruña, Spain; alberto.garcia.fernandez@udc.es (A.G.-F.); jorge.salgado@udc.es (J.S.-B.); m.andujar@udc.es (M.S.-A.); 5 Coordenação de Ciências Naturais, Universidade Federal do Maranhão, Campus do Bacabal, São Luís 65700-000, Brazil

**Keywords:** azide frameworks, hybrid perovskite, Raman spectroscopy, phase transitions

## Abstract

Temperature-dependent Raman scattering and differential scanning calorimetry were applied to the study of the hybrid organic-inorganic azide-perovskite [(CH_3_)_4_N][Cd(N_3_)_3_], a compound with multiple structural phase transitions as a function of temperature. A significant entropy variation was observed associated to such phase transitions, |∆S| ~ 62.09 J·kg^−1^ K^−1^, together with both a positive high barocaloric (BC) coefficient |δT_t_/δP| ~ 12.39 K kbar^−1^ and an inverse barocaloric (BC) coefficient |δT_t_/δP| ~ −6.52 kbar^−1^, features that render this compound interesting for barocaloric applications. As for the obtained Raman spectra, they revealed that molecular vibrations associated to the NC_4,_ N_3_^–^ and CH_3_ molecular groups exhibit clear anomalies during the phase transitions, which include *splits* and discontinuity in the phonon wavenumber and lifetime. Furthermore, variation of the TMA^+^ and N_3_^–^ modes with temperature revealed that while some modes follow the conventional red shift upon heating, others exhibit an unconventional blue shift, a result which was related to the weakening of the intermolecular interactions between the TMA (tetramethylammonium) cations and the azide ligands and the concomitant strengthening of the intramolecular bondings. Therefore, these studies show that Raman spectroscopy is a powerful tool to gain information about phase transitions, structures and intermolecular interactions between the A-cation and the framework, even in complex hybrid organic-inorganic perovskites with highly disordered phases.

## 1. Introduction

Compounds that combine simultaneously organic and inorganic chemical groups are of great interest since they enlarge the range of structural possibilities that allow the coexistence and modulation of fundamental physical properties, increasing their multifunctional potential [1]. These hybrid inorganic-organic compounds have attracted great attention in the last years in view of their high technological potential in the areas of optoelectronics [2], photovoltaics [3], ferroelectrics [4], multiferroics [5,6], and, very recently, also in the field of barocaloric materials for solid-state cooling [7,8].

In this context, the ABX_3_ perovskite family—which, for decades, has played a prominent role in the evolution and application of inorganic materials—has experienced a large expansion by incorporating organic blocks in its structure, giving rise to very outstanding materials known as hybrid perovskites. In these relatively new compounds, the A site is usually occupied by an organic cation (A = protonated amine), the B site may be occupied by a divalent transition metal cation (e.g., B = Mn^2+^, Co^2+^, Ni^2+^, Cd^2+^, etc.) and the X site by a monatomic anion, mainly a halide, such as Cl^−^, Br^−^ and I^–^, or anionic polyatomic bridge ligands—for instance, azides (N_3_^–^), cyanides (CN^–^) and formates (HCOO^–^) [1,9,10,11].

The introduction of elongated bridge ligands in the X site allows the building of frameworks that can accommodate a high variety of larger organic ions in the A site, giving rise to structures that are, in general, more flexible and susceptible to phase transformations as compared to those built using shorter X [12,13]. In this context, hybrid perovskites with X = azide (N_3_^−^) and general formula [(CH_3_)_n_NH_4−n_][M (N_3_)_3_], n = 1–4, where M: divalent metallic cation or bimetallic combinations of M^3+^ M^+^ are very attractive in view of their interesting ferroic properties [6] and extensive structural transitions [11,14,15]. In these materials where the azide groups act as end-to-end (EE, µ-1,3-N_3_) almost linear bridging ligands, magnetic coupling between metallic centers is also more favored than in hybrids with other polyatomic ligands, so azide perovskites typically exhibit higher magnetic transition temperatures than other analogous hybrids, such as formate perovskites. [16,17].

A very prominent member of this family is the manganese azide with A: [(CH_3_)_4_N]^+^ (n = 4), (TMA), (TMAMnN_3_), the first azide reported to have a three-dimensional framework structure [17], which is now known to show coexistence of three ferroic orders (antiferroelectric, ferroelastic and magnetic bistability) [6,16]. In addition, in view of its very large entropy change at the ferroelastic phase transition (two times larger than in the case of the giant barocaloric hybrid perovskite [TPrA][Mn(dca)_3_] (TPrA = (CH_3_CH_2_CH_2_)_4_N^+^, dca = [N(CN)_2_]^−^) [8]), it has been first proposed [6] and recently experimentally proved that this material is a very promising barocaloric material [18].

**Figure 1:**
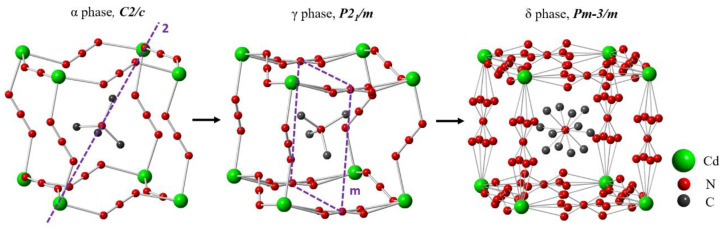
Perspective view of the crystal structures of the α, γ and δ phases of [(CH_3_)_4_N][Cd(N_3_)_3_]. Cd, N and C atoms are shaded in green, red and dark gray, respectively.

By replacing B: Mn^2+^ with Cd^2+^, the Cd-azide perovskite [(CH_3_)_4_N][Cd(N_3_)_3_] (TMACdN_3_) is obtained, which can be described as consisting of three-dimensional anionic cages [Cd(N_3_)_3_]*⁻* containing TMA as A-cation [19]. This compound is even more susceptible to structural transformations than the Mn-azide as it exhibits three reversible first-order structural phase transitions as a function of temperature (from phase α to phase β (at T = 270 K), from phase β to phase γ at T = 277 K and from phase γ to phase δ at T = 322 K). According to the literature, such transformations imply order-disorder processes related mainly to the sway of the conjugated N=N=N bonds*,* as well as tetrahedron rotations of the A-cation [(CH_3_)_4_N]^+^ [15]. In this context, and according to the literature, at temperatures up to 270 K, the crystal structure of this compound is a centrosymmetric space group *C*2/*c* (No. 15, Z = 4) (α-phase). As it can be seen in Figure 1, in this polymorph, the [Cd(N_3_)_3_]^−^ framework is considerably distorted, the azide ligands—which are slightly bent—are completely ordered and the TMA^+^ cations are centered in the A-sites. There is also a cooperative tilting of the [CdN_6_] octahedra. At 270 K, on heating, this polymorph transforms into a β phase, with a narrow stability range of only 7 K that hinders its structural characterization [15]. Upon further heating, at 277 K, this polymorph transforms into the γ-phase, which is monoclinic, with space group *P*2_1_/*m*. This polymorph is characterized by a subtle disorder in the slightly bent azide ligands along the direction [010], as shown in Figure 1, where the two N atoms (N3, N3′) that bind the Cd ions alternate between two crystallographic positions (Wyckoff site 4f), while the third one (N4) occupies a unique site (4e). Meanwhile, the other N_3_^−^ bridges are ordered and completely linear. Another interesting feature is the considerable distortion of the [Cd(N_3_)_3_]^−^ framework, with an also slightly distorted octahedral environment for the Cd^2+^ cations (three different Cd-N distances) and an unconventional cooperative tilting of such octahedra, where adjacent octahedra display a cooperative in-phase rotation within the ac plane (all of them are rotated either clockwise or counter-clockwise), while along the b-axis, adjacent octahedra are oppositely rotated (alternating clockwise and counter-clockwise rotations); see Figure S2 of the supplementary materials. This unconventional tilting cannot exist in pure inorganic ABO_3_ perovskites.

Another interesting feature of this polymorph is the off-center shift of the TMA from the center of the cavities.

At temperatures above 322 K, TMACdN_3_ transforms into a cubic phase (δ-phase), belonging to the *Pm*-3*m* space group (No. 221, Z = 1) [15], characterized by a large structural disorder of the azide ligands, where the rod-like N_3_ oscillates among four sites. Another significant feature is the disorder in TMA^+^, where the four (4) positions of the carbon ions in TMA^+^ cation unfold into 12 positions, as illustrated in Figure 1.

In this paper, we try to gain more insight into such phase transformations and in the role of interactions between the A-cation and the framework in such structural transitions, an aspect that has not been analyzed so far in this compound. For this purpose, we use Raman spectroscopy as a tool that can be very powerful to study the mechanisms of structural phase transitions, to detect effects of order-disorder and to clarify how symmetry breaks; specifically, which vibrations, ions and molecular arrangements are strongly related to the given phase transitions [20,21,22,23,24,25,26,27]. Furthermore, from differential scanning calorimetric (DSC) measurements and structural data available in the literature, we estimate the barocaloric coefficients (|δT_t_/δP|) and entropy changes for each of these transitions to evaluate the potential of this compound as barocaloric material.

## 2. Results and Discussion

### 2.1. Basic Characterization and Deeper Insight into the Crystal Structure of TMACdN_3_

Room-temperature experimental X-ray powder diffraction results confirmed that the obtained sample of TMACdN_3_ is single phase as no impurities were present, and that at this temperature, it exhibits the expected crystal structure for the γ-phase. Details of the comparison between the experimental X-ray powder diffraction pattern of TMACdN_3_ at room temperature and the simulated X-ray diffraction pattern from single crystal measurements available in the literature [15] are given in the Supporting Information (see Figure S1).

On the other hand, to gain more insight into the phase transitions, we analyzed, in detail, the intermolecular interactions between the TMA cation and the [Cd(N_3_)_3_]^−^ framework of the different polymorphs on the basis of the Hirshfeld surface analysis; see Fig. S3 of SI. This analysis shows that there are interactions between the H atoms of the TMA cation and the N atoms of the azide ligands (red regions at the Hirshfeld surface) both in the α- and γ-polymorphs. In addition, we observed that there are differences between the number of azide ligands involved in these interactions in each of these polymorphs. In the case of α-phase, the azide ligands (except the four along the a-axis) are all involved in the link with the TMA cation. Meanwhile, only two azide ligands (those located along the b-axis) are involved in such interactions in the case of the γ-phase. It is worth noting that these two different situations are also related to the location of the TMA inside of the pseudocuboctahedral cavity. In this context, as in the α-phase, the TMA cation is located at the center of the cavity and most of the azide ligands can interact with that cation. In contrast, in the case of the γ-phase, where the TMA cation is shifted from center of the cavity towards two of the azide ligands, those N_3_^−^ are the only ones that can interact with it.

During the discussion of the Raman results, we will show how these interactions between the TMA cation and the azide ligands, which are strongly influenced by temperature change, especially in the phase transition regions, are reflected and can be followed through the Raman spectra profile.

### 2.2. Thermal Characterization (DSC) and Barocaloric Parameters

**Figure 2:**
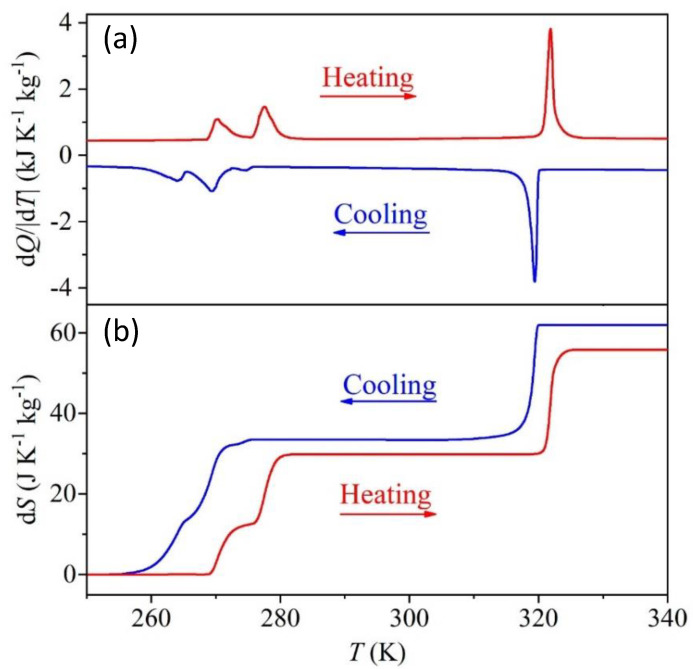
(**a**) Differential scanning calorimetric (DSC) results as a function of temperature obtained by heating and cooling the TMACdN_3_ compound. (**b**) Isobaric entropy change as a function of temperature due to the first-order phase transitions α→β→γ and γ→δ (obtained from experiments performed on heating and cooling).

DSC measurements confirm that the compound undergoes three reversible structural phase transitions as a function of temperature with T_heating_/T_cooling_ = 270/263, 277/270 and 322/319 K, with an overlap of the peaks of the first two transitions (α→β and β→γ) and a sharp peak regarding the ferroelastic transition (γ→δ) (see Figure 2a). From the area under the peaks, we have obtained the isobaric enthalpy change ∆*H_ib_* for the α→β→γ transitions, which were analyzed jointly, and ferroelastic transition; see obtained values in Table 1. Additionally, we have also calculated the isobaric entropy change ∆S_ib_ as a function of temperature using the following relation $∆S_{ib}=\int_{T_{0}}^{T}\frac{1}{T}\frac{Q\left(P\right)}{T’}dT$, where Q(P) is the heat flow measured at constant pressure, $T^{\prime }$ is the temperature rate and T is the temperature, as it is shown in Figure 2b. The isobaric entropy change as a function of temperature grows abruptly until it reaches a local maximum plateau of ~29.83 J·Kg^−1^K^−1^ at the transition α→β→γ and ~32.26 J·Kg^−1^K^−1^ at the ferroelastic transition. Therefore, the total entropy change for the three phase transitions turns to be 62.09 J·kg^−1^ K^−1^, which is in excellent agreement with the value reported in the literature [15,26].

As in order-disorder phase transitions, ∆S is given by R ln(N) with *N* = (*n*_2_/*n*_1_), where *n_2_* and *n_1_* are the number of configurations in each polymorph and R is the gas constant (8.314 J mol^−1^ K^−1^), we have estimated, from the heating data, an N = 2.9 and 2.5 for the α→β→γ transition and ferroelastic, respectively. All the values of enthalpy change ∆H, entropy change ∆S and N under heating and cooling are summarized in Table 1.

Additionally, following the procedure reported in the literature [7], we have estimated different barocaloric parameters to evaluate the potential of this hybrid perovskite as a barocaloric material.

In this context we have to note that TMACdN_3_ shows relevant features, which, in principle, render it a good candidate to show high barocaloric effects, especially as it has a relative large reversible ferroelastic structural transition, whose critical temperature is close to room temperature, making it highly desirable for practical applications; the intrinsic flexibility of the azide ligand, which is part of the framework, makes it susceptible to large volume variations under applied external pressure.

To estimate the barocaloric (BC) coefficient of TMACdN_3_, we have used the Clausius-Clapeyron method, which is a widely used indirect method in the case of caloric materials [7]. Taking into account the following expression, (δTt/δP) = (Δ*v*/∆S), where Δ*v* is the volume change at the phase transition and ∆S is the entropy change of the phase transition, we calculate the BC coefficient from our calorimetric results (on heating) as well the structural data (volume) in the vicinity of the structural transition available in the literature [28]. Following this method, we have estimated the barocaloric coefficient for the α→β→γ phase transition and for γ→δ, as shown in Table 1. As it can be seen there, the two barocaloric coefficients are very different, not only in magnitude (one almost double than the other) but also in sign. In this context, while the α→β→γ phase transition exhibits a positive, conventional BC coefficient (which means that the γ phase heats up when adiabatically squeezed and cools down when pressure is released close to this phase transition temperature), the ferroelastic γ→δ transition displays a negative, that is, inverse, BC coefficient (that is, the γ phase cools down when pressure is applied and heats up when it is released close to the ferroelastic phase transition temperature).

For the α→β→γ phase transitions, the BC coefficient is 12.39 K kbar^−1^, which is similar to that exhibited by the related azide hybrid perovskite TMAMnN_3_ [18]. In any case, it is worth to note that the BC coefficient of TMACdN_3_ is very large in comparison with BC inorganic compounds (such as alloys and oxides), which typically exhibit values inferior to 1 K kbar^−1^ [29]. Very interestingly, the ferroelastic transition displays an inverse BC coefficient, which is very scarce, and few materials are known with this property. Therefore, the BC parameters indicate that TMACdN_3_ is an interesting material from BC applications with an adequate working temperature, close to room temperature between 260 and 320 K, and isobaric entropy change values almost similar to those reported for related [TPrA][Mn(dca)_3_] hybrid perovskite, whose values is 38.1 J·Kg^−1^K^−1^ (until now the highest report value for a BC hybrid perovskite).

### 2.3. Raman Studies

#### 2.3.1. Room Temperature Raman Spectrum

**Figure 3:**
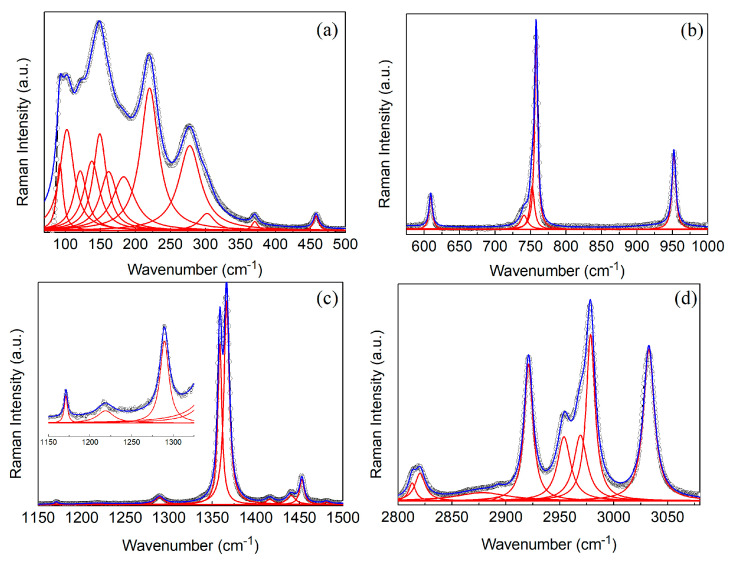
(**a**–**d**) show Raman spectra of TMACdN_3_ measured at room temperature in different spectral regions. The open circles are the experimental data, while the blue line is the best fit employing Lorentzian’s function. The inset at figure (**c**) shows a zoom of the medium-wavenumber region of the spectrum.

Figure 3 shows the Raman spectrum obtained for TMACdN_3_ at room temperature, which, in fact, is rather similar to that observed for TMAMnN_3_ and DMAMnN_3_ [24] compounds. According to group theory, considering the irreducible representations of the group factor $C_{2h}$ (2/*m*) and the occupations of the Wyckoff sites of the space group $C_{2h}{}^{2}$ (*P*2_1_/*m*), 72 Raman active modes are predicted ($\Gamma^{Raman}=\;38\;A_{g}⊕34\;B_{g}$). Among those, 33 modes were observed, being a reasonable number considering that in these compounds, a large grouping of modes is expected in narrow bands of the spectrum. The vibrational modes investigated are mainly attributed to internal vibrations of the TMA^+^ cations, azide anions and lattice vibrations [24]. Most internal modes of TMA^+^ cations and azide ligands are observed in distinct regions of the spectrum, which facilitates the assignment and comparison with similar compound spectra. Thus, our assignment of the observed modes was based on Raman investigations of similar compounds available in literature [23,29,30,31] and it is summarized in Table 2.

As can be seen there, at frequencies below 300 cm^−1^, lattice modes are mainly observed and include translational and librational modes of the TMA^+^ cations, N_3_^−^ organic groups as well as those of Cd^2+^ ions. In this region, less pure modes are also observed, with quite representative intensities, such those at 220 and 274 cm^−1^, which can be classified as N_3_^−^ librations and a combination of CH_3_ group twisting with an Cd ion translation, respectively.

The intermediate- and high-frequency regions are dominated by internal modes. A rather prominent mode attributed to the symmetric stretching of the NC_4_ group is observed at approximately 758 cm^−1^. The region between 1000–1500 cm^−1^ exhibits several low-intensity modes mainly due to CH_3_ rocking (ρ modes) observed at about 1047, 1171, 1218 and 1359 cm^−1^ and δ-bending (scissoring) of CH_3_ group (δ_as_CH_3_ δ_s_CH_3_) as the mode at 1453 cm^−1^. This band also includes the azide group symmetric stretching ($\nu_{1}$ mode) at 1359 cm^−1^, which is a very intense and important mode for monitoring the azide ligands N_3_^−^ (see Table 2).

In the higher-frequency region, above 2000 cm^−1^, internal modes related to TMA^+^ prevail, such as the symmetric stretching of the CH_3_ group and combinations of symmetric stretching and asymmetric stretching of this methyl group. As we will show, these medium-intensity modes are very sensitive to disorder and modifications in the chemical environment of TMA during phase transitions. In this region, we highlight the 2953 and 2978 cm^−1^ modes that are attributed to pure CH_3_ symmetric stretching vibrations and those observed at 2921 and 3032 cm^−1^ that correspond to mixed symmetric and asymmetric stretching modes of the CH_3_ group.

#### 2.3.2. Raman Spectra as a Function of Temperature

The factor group analysis of the fundamental modes and correlation diagram for the low temperature α-phase (space group *C*2/*c*) are presented in Table S1 of supplementary materials. Those of the γ-phase would be similar to the ones already published by M. Trzebiatowska for the TMMnN_3_ compound [24]. Furthermore, since the δ-phase of TMCdN_3_ is highly disordered, its analysis is not given here.

**Figure 4:**
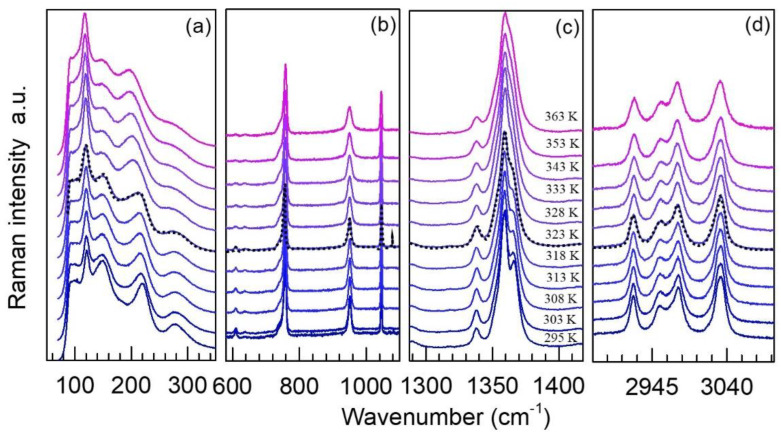
Representative Raman spectra of TMACdN_3_ at selected temperatures in the region of (**a**) 30–350 cm^−1^, (**b**) 580–1110 cm^−1^, (**c**) 1287–1418cm^−1^ and (**d**) 2901–3000 cm^−1^, normalized to the intensity of the highest peak and shifted vertically for better visualization and comparison. The dashed black line indicates the first-order (γ→δ) structural phase transition. In the other regions of the spectral range, as shown in Figure 5b,d, more subtle changes may be noticed and will be discussed below in more detail.

**Figure 5:**
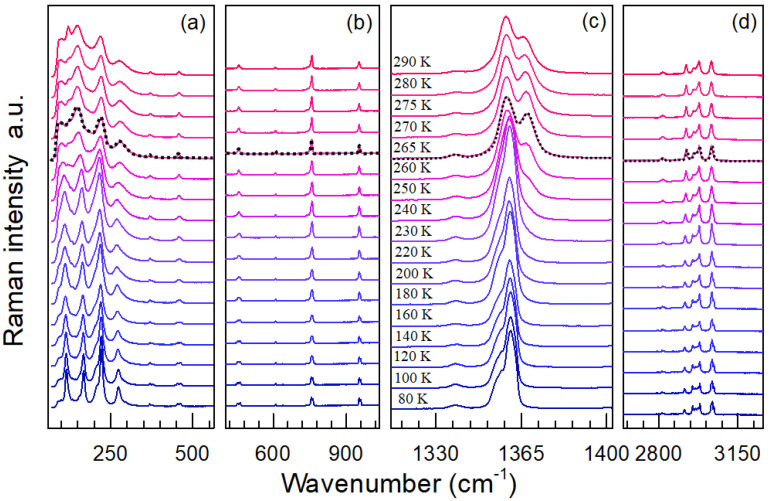
Representative Raman spectra of TMACdN_3_ at selected temperatures in the region of (**a**) 64–564 cm^−1^, (**b**) 403–1035 cm^−1^, (**c**) 1312–1402 cm^−1^ and (**d**) 2641–3262 cm^−1^. The dashed black line indicates the first-order α→β→γ structural phase transition.

Figure 4 and Figure 5 show representative normalized Raman spectra of TMACdN_3_ from 295 K to 365 K in the frequency range 30 to 3100 cm^−1^ and from 80 K to 290 K in the range 50 to 3300 cm^−1^, respectively. In both cases, the dashed lines indicate the critical temperatures for the phase transitions according to DSC, T_γ__→δ(heating)_ = 322 K (Figure 5) and T_γ__→β__→α(cooling)_ = 265 K, temperatures at which significant changes in the Raman spectra also occur (see below).

As shown in Figure 4 and Figure 5, the main changes observed in these spectra as temperature increases can be summarized as follows: in first place, a broadening of the modes and, in general, a decrease in their intensity—see, for example, Figure 5a. Especially interesting is the region of 1312−1402 cm^−1^, corresponding to the azide group ν_s_ modes (ν_1_), where the band, which is split at low temperatures, seems to merge into one at T(γ  α), while a new shoulder at 1366 cm^−1^ starts to develop in the vicinity of this critical temperature; see Figure 5c. For higher temperatures (Figure 4c), a new approximation of the azide group ν_s_ modes (ν_1_) at 1359 and 1366 cm^−1^ takes place, finally giving rise to a broad band.

**Figure 6:**
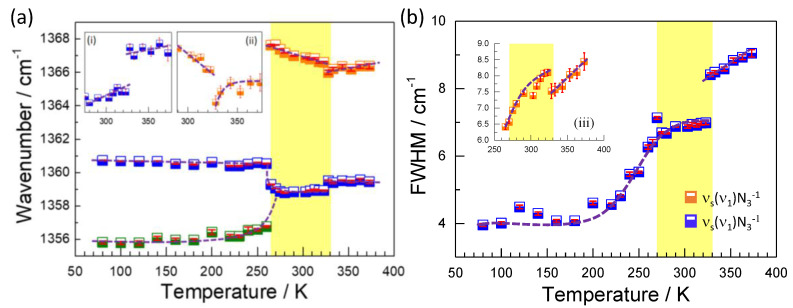
Temperature dependence of the wavenumber (**a**) and full width at half-maximum (FWHM) (**b**) of the most representative modes in the region of medium frequencies. The insets (i) and (ii) indicate details of the wavenumber behavior around the second structural phase transition; meanwhile, the inset (iii) indicates details of the multiple phase transitions observed in the width of the mode 1366 cm^−1^.

**Figure 7:**
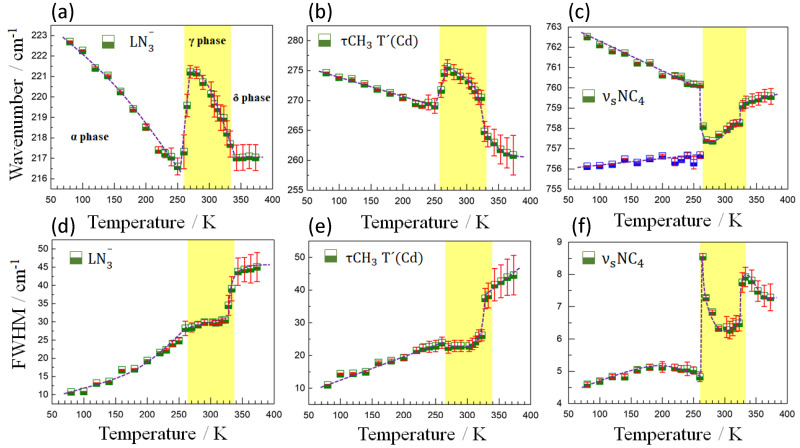
Temperature evolution of the wavenumber (**a**–**c**) and FWHM (**d**–**f**) of the most representative modes in the region of lower frequencies. The half-filled blue and green squares represent experimental data and the solid dotted purple lines represent eye guidelines. The solid vertical lines in red indicate the calculation error bar.

In order to perform a more detailed analysis of the behavior of the phonons during the temperature-induced multiple structural phase transitions in TMACdN_3_, we show, in Figure 6 and Figure 7, the behavior of the most intense modes which were more susceptible to structural changes. In addition, we also include the temperature dependence of their full width at half-maximum (FWHM), which depends on the phonon’s lifetime in the lattice and their anharmonicities. As it is well-known, FWHM is very sensitive to structural disorder, whose presence contributes to reducing the phonon’s lifetime and consequently increases the width of the spectral bands [32,33,34,35].

Figure 6 shows the behavior of the wavenumber and FWHM of the modes related to the azide group which can be attributed mainly to the symmetrical stretching vibrations ν_s_(ν_1_)N_3_^−^. In particular, the very intense band at 1359 cm^−1^ is presented as the main mode because it remains present, with slight modifications, in all structural phases. As qualitatively explained before, for T < 265 K in the low-temperature phase, a splitting is observed and a new mode emerges at 1355 cm^−1^, while the main mode experiences a red shift to 1361 cm^−1^. For 265 < T(K) < 323, the main mode experiences a blue shift back to 1359 cm^−1^ and a new band appears at 1366 cm^−1^, showing the α→γ phase transition. In addition, discontinuities in the phonon energy at 1359 and 1366 cm^−1^ are observed at approximately 323 K (under heating), where the second structural phase transition γ→δ occurs. A strong narrowing and discontinuity in the width of the modes 1359 and 1366 cm^−1^ are also observed at 323 K; see Figure 6.

We rationalize the observed behavior as follows: at low temperatures, below 265 K, the splitting of the observed symmetrical stretching vibrations ν_s_(ν_1_)N_3_^−^ reflects the presence of two groups of azide ligands, which mainly differ in the degree of interaction with the H atoms of the TMA cation. Those with stronger azide-H-TMA interactions (and thus with more weakened and more enlarged intraligand N-N bonds) give rise to a lower wavenumber, while those that do not interact with the TMA cation through the H atoms (and thus with stronger N-N intraazide bonds) give rise to higher wavenumber bands; see Figure S4 of supplementary materials. The assignment of azide ligands is reinforced by the temperature dependence of both ligands. The one with stronger interactions shows an increase of wavenumber on heating, which is an anomalous behavior, due to the weakening of this interaction upon heating.

At 265 K, the structural transformation and the concomitant changes in the distances and angles in the azide-framework interaction result in the breaking of H bonds between the azides that were initially interacting with the framework. Furthermore, a majority of azides get liberated from such bonding and strengthen their intraligand N-N bonds, giving rise to the appearance of a higher number shoulder (about 1367 cm^−1^); see Figure S4 of supplementary materials. We suggest that the off-center shift of the TMA seems to play an important role at the observed large splitting of the azide group symmetrical stretching vibrations. Again, the temperature dependence of both modes upon heating is in agreement with the proposed assignment. The non-interacting azide ligands exhibit a red shift on heating due to the weakening of N-N bond interactions. In contrast, the interacting azide ligand shows a blue shift on heating due to the weakening of the intermolecular interaction and the stronger intramolecular bonding.

Above T > 332 K, the dynamic forming and breaking of much weaker azide-H-TMA bonds could be the reason for the broad bands observed above that critical temperature.

Furthermore, the strong change observed in the width of the modes could be reflecting variations in the degree of structural disorder in the azide ligands, similar to that observed for TMAMnN_3_ and NaN_3_ [24,36] crystals. Furthermore, the variation in the width of the mode observed at 1359 cm^−1^ during the γ→δ phase transition is abrupt, with a discontinuity, which is, in fact, consistent with the strong increase in the disorder in the azide ligands in the δ phase.

It should be noted that these results differ significantly from those obtained in the Mn azide [24], where a much broader Raman band for the symmetrical stretching vibration of the azide ligand did not allow to see these changes as a function of temperature.

On the other hand, Figure 7 shows the behavior of the wavenumber and FWHM of the modes observed at 220, 274 and 758 cm^−1^ for the entire temperature range studied. Through the anomalies observed in the behavior of these modes, we can also clearly identify the two phase transitions (α→γ and γ→δ) occurring at approximately 265 K (under cooling) and 323 K (under heating), both temperatures being in excellent agreement with the DSC measurements. As for the first phase transition (α→γ), it is observed that the modes at 220 and 274 cm^−1^ follow a natural softening behavior with an increase in temperature, to subsequently suffer an abrupt increase in energy, an anomalous blue shift of ~4cm^−1^, followed by a new softening trend after the transition. Such variations can be mainly attributed to structural changes perceived in the LN_3_^−^ and τCH_3_ T′(Cd) vibrations, respectively, probably related to the cooperative tilting of the [CdN_6_] octahedra and concomitant framework distortion that occur at that temperature. As for the behavior of the FWHM of these modes, a sudden broadening is observed with increasing temperature, in agreement with the slight order-disorder effect in the azide ligand in the intermediate phase.

Regarding the γ→δ transition, an abrupt variation in the width of the modes at 220 and 274 cm^−1^, which undergo an increase in width of ~15 cm^−1^, is observed in contrast to the variation of the same modes at the α→γ transition. This clearly demonstrates a direct relationship between the FWHM change and the degree of structural disorder since the δ phase exhibits high structural disorder in the TMA cations and in all crystallographic directions for the N_3_^−^ ligand. On the other hand, the anomalies observed in the wavenumber were more subtle.

Figure 7 also shows the temperature dependence of the wavenumber and FWHM of the mode at 758 cm^−1^, characteristic for the NC_4_ group (TMA), which is split into two modes at temperatures below 265 K as a result of symmetry reduction and merges into a single one above that critical temperature. It is important to highlight that during the α→γ transition, the width of this mode is characterized by a strong discontinuity, at difference with the behaviors observed for the widths of the modes at 220 and 274 cm^−1^. This indicates that short-range disorder effects on TMA cations must be present in the α→γ transition since the long-range subtle structural changes could not justify such a significant variation in FWHM. Furthermore, during the γ→δ phase transition, an anomalous hardening of this mode is observed with increasing temperature, behavior which can be explained by the shortening of the C-N bonds (from 1.486 to 1.492 Å in the α-phase to 1.465 to 1.486 Å in the γ-phase and 1.411 Å in δ phase [14]), probably related to a strengthening of the N-C bond upon weakening of the TMA-azide interaction.

**Figure 8:**
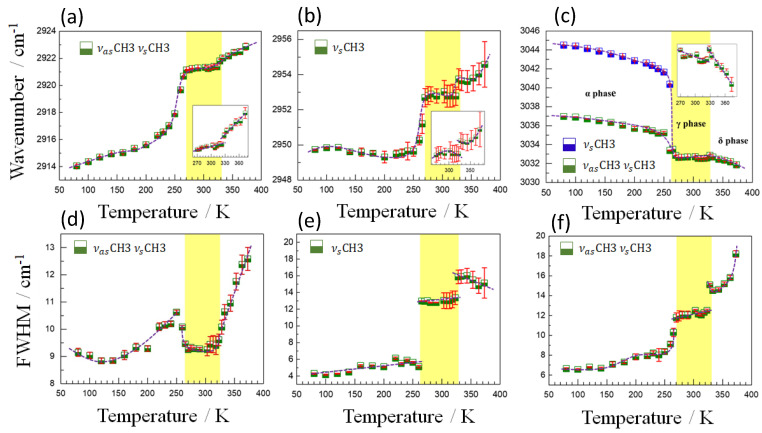
Temperature dependence of the wavenumber (**a**–**c**) and FWHM (**d**–**f**) of the most representative modes in the region of higher frequencies. The half-filled blue and green squares represent experimental data and the solid dotted purple lines represent eye guidelines. The solid vertical lines in red indicate the calculation error bar. The insets (**a**–**c**) indicate details of the wavenumber behavior around the second structural phase transition.

Finally, Figure 8 shows the temperature dependence of the wavenumber and FWHM of the modes associated with the CH_3_ group observed at 2921, 2952 and 3032 cm^−1^. As it can be seen, the frequency of the 2921 and 2952 cm^−1^ modes has a very similar behavior with temperature, characterized by the anomalous hardening of these modes as temperature increases. Meanwhile, those at higher frequencies (3032 cm^−1^) follow the expected behavior as a function of temperature, even if with a sharp jump at the phase transitions.

As for the 2921 and 2952 cm^−1^ modes, their anomalous behavior is probably related to the fact that they correspond to TMA cations that are interacting with the azides in the framework. In this case, the data reveal a strengthening of the intraatomic C-H bond as temperature increases and the azide-H-TMA interaction decreases. In addition, both modes exhibit anomalies that can be easily identified during the α→γ and γ→δ phase transitions. The first anomaly, observed at approximately 323 K, is characterized by an attenuation in the tendency of softening of the modes with temperature reduction. After the second transition at ~265 K (under cooling), the softening of these modes becomes quite pronounced in good agreement with the changes commented in the azides related to the azide-H-TMA interaction. Such anomalies can also be observed in the width of these modes, which show prominent changes at ~265 K and 323 K. In particular, the width of the mode 2952 cm^−1^ undergoes a strong discontinuity (~8 cm^−1^) at approximately 265 K, which can be associated with a structural disorder of the CH_3_ group, marking the first transition. Above 323 K, a discontinuity in the width of this mode reveals that the second transition is strongly influenced by the disorder effects of the CH_3_ group. Regarding the band observed at 3032 cm^−1^, a splitting of modes occurs at temperatures below 265 K and a slight change in the trend in the wavenumber during the ferroelastic transition. The width of the mode at 3032 cm^−1^ also presents clear anomalies near to critical temperatures, proving the structural transitions. Interestingly, the anomalies observed in FWHM in these last three modes related to TMA reinforce that short-range disorder effects are present during the α→γ structural phase transition and are perceived in the vibrations of the CH_3_ and NC_4_ groups, as previously observed in Figure 7. Variations in the short-range configuration certainly contribute to the experimental N value (∆S = R ln (N)) during the α→γ transition, showing higher values than those observed for the ferroelastic transition (see Table 1).

## 3. Materials and Methods

### 3.1. Synthesis

Block-shaped single crystals of TMACdN_3_ were obtained by the slow evaporation method as previously reported [15]. An aqueous solution (10 mL) of NaN_3_ (390 mg, 6 mmol) and (CH₃)₄NCl (630 mg, 3 mmol) was mixed with an aqueous solution (5 mL) of Cd(NO_3_) 4H_2_O (154 mg, 0.5 mmol). The resulting solution was filtered through a sieve (0.22 μm) and the obtained clear solution was kept at room temperature. After 3 days, transparent crystals were observed at the bottom of the glass.

### 3.2. Powder X-Ray Diffraction

Powder X-ray diffraction (PXRD) patterns of the obtained powders and of grounded single-crystals were collected in a Siemens D-5000 diffractometer (Aubrey, TX, USA) using Cu K_α_ radiation at room temperature.

### 3.3. Hirshfeld Surface Analysis

Identification of close contacts between the framework and the TMA cations in the cavities was carried out by means of Hirshfeld surface analysis using CIF (Crystallographic Information Framework) data [15] and the CrystalExplorer 17.5 software [37].

### 3.4. Differential Scanning Calorimetry—DSC

Differential scanning calorimetric (DSC) analyses were carried out in a TA Instruments DSC Q-2000 (Waters, Cerdanyola del Valles, Spain) by heating and cooling the samples under a nitrogen atmosphere, during several cycles at 10 K/min.

### 3.5. Temperature-Dependent Raman Spectroscopy

The temperature-dependent Raman measurements were carried out in the 80–373 K range using a Horiba Jobin-Yvon T64000 triple-grating spectrometer (Horiba/Jobin Yvon/ISA, Edison, NJ, USA). For the high- and low-temperature measurements, a Linkam TS1200 heating stage and a CTI-Cryogenic M-22 closed-cycle He refrigerator system were used, respectively. A 532.0 nm radiation from a Diode-Pumped Solid-State Laser (DPSSL) (Horiba/Jobin Yvon/ISA, Edison, NJ, USA), operating at ~14 mW, was used as the excitation source. The spectra were collected in back-scattering geometry with a resolution of 2 cm^−1^ on heating, in the case of the high T measurements, and upon cooling, in the case of the low T measurements. An Olympus BX41 microscope equipped with a 20× long working distance (WD = 20.4 mm) objective lens was used to focus the laser beam on the sample surface (Olympus, Center Valley, PA, USA), and the Raman signal was detected with an N2-cooled Charge-Coupled Device (CCD) (Olympus, Center Valley, PA, USA).

## 4. Conclusions

Crystals of the azide compound [N(CH_3_)_4_][Cd(N_3_)_3_] belonging to the hybrid organic-inorganic perovskite family were obtained by the slow evaporation method. DSC measurements demonstrated that the compound experiences multiple structural transitions, with a total entropy change of |∆S| ~ 62.09 J·kg^−1^ K^−1^. The estimated barocaloric coefficient, (δT_t_/δP), gives values of 12.39 and −6.52 K kbar^−1^ for the α→β→γ and the ferroelastic phase transitions, respectively. These values are very large in comparison with BC inorganic compounds (such as alloys and oxides) and similar to those found in the analogous TMAMnN_3_ hybrid perovskite [18]. Very interestingly, the ferroelastic transition displays an inverse BC coefficient, which is very scarce, and few materials are known to exhibit this behavior. In addition, its working temperature is close to room temperature, between 260 and 320 K. All these findings indicate that TMACdN_3_ is an interesting material for BC applications.

On the other hand, a detailed study of the temperature dependence of Raman modes between 80 and 373 K was carried out. In this context, the internal vibration groups of the TMA cation and the N_3_^−^ azide ligand and the lattice vibrations were distinguished in specific spectral bands, allowing classifications of the modes and individualized monitoring of the vibrations by molecular groups as a function of temperature. In the vicinity of the critical temperatures of the α→γ and γ→δ transitions, the vibrational frequencies and FWHMs exhibited clear anomalies, indicating the onset of the first-order structural phase transitions. From analysis of the variation of TMA and azide modes with temperature, it was observed that many modes follow the conventional red shift upon heating, while other modes exhibit an unconventional blue shift, which were related to the weakening of intermolecular interactions and the strengthening of intramolecular bonding, respectively.

Abrupt variations in the width of the modes related to TMA^+^, particularly in the vibrations of symmetric and asymmetric stretching of CH_3_ molecular group and the symmetric stretching of the NC_4_ group, indicate that short-range disorder effects are present during the α→γ structural transition.

These results show that Raman spectroscopy is a powerful tool to gain information about phase transitions and intermolecular interactions between the A-cation and the framework, even at disordered phases, in complex hybrid organic-inorganic perovskites.

## Figures and Tables

**Table 1:** Summary of thermodynamic parameters for the phase transitions of TMACdN_3_ compound obtained from DSC analysis under heating and cooling. *|*∆*H_ib_|* is the isobaric enthalpy change, *|*∆*S_ib_|* is the isobaric entropy change, N is the number of sites in the disordered phase and *δT_t_/δP* is the barocaloric coefficient.

Parameters	Heating		Cooling	
	T_α__→β_/T_β__→γ_	T_γ__→δ_	T_α__←β_/T_β__←γ_	T_γ__←δ_
*T_t_* (K)	270.4/277.6	322.0	263.9/269.6	319.5
|∆*H_ib_*| (J·g^−1^)	8.40	8.46	9.22	9.14
|∆*S_ib_*| (J·Kg^−1^K^−1^)	29.83	25.97	32.26	28.51
N	2.9	2.5	3.2	2.8
*δT_t_/δP* (K kbar^−1^)	12.39	−6.52	-	-

**Table 2:** Observed Raman modes (in cm^−1^) of TMACdN_3_ and their respective assignment.

C2/c (80 K)	P2_1_/m (298 K)	Pm-3m (373 K)	Assignment
3044 m			ν_s_CH_3_
3037 s	3032 m	3031m	ν_as_CH_3_ ν_s_CH_3_
2982 s	2978 m	2978 m	ν_s_CH_3_
2972 m	2969 sh	2970 sh	ν_as_CH_3_ ν_s_CH_3_
2961 m/2949 s	2953 m	2954	ν_s_CH_3_
2917 vw	2921 m	2923 m	ν_as_CH_3_ ν_s_CH_3_
2821 vw	2820 w	2824 w	2xδ_as_CH_3_
2812 vw	2813 w	2814 vw	2xν_s_(ν_1_)N_3_^−^
1479 vw	1481 vw	1471 vw	δ_as_CH_3_ δ_s_CH_3_
1462 vw	1453 w	1451 w	δ_as_CH_3_ δ_s_CH_3_
1454 s/1438 s/1418 vw	1440 vw	1440 vw/	combination
1399 vw			combination
	1366 vs	1367 s	ν_s_(ν_1_)N_3_^−^
1360 vs/135 5m	1359 vs	1359 vs/	ν_s_(ν_1_)N_3_
1338 w	1337 vw	1337 w	
1290 m	1289 w	1285 vw	ρCH_3_
1273 w		1267 vw	ρCH_3_
1217 w	1218 vw		ρCH_3_
1179 w	1171 vw	1174	ρCH_3_
1170 w			ρCH_3_
1045 w	1047 vw	1043 w/1047 vw	ρCH_3_
958 m/952 m	952 w	950 w	ν_as_NC_4_
762 m	758 m	759 m	ν_s_NC_4_
755 m	752 sh	753 w-sh	
730 vw	741 vw	741 vw	
609 w	609 w		
463 w/453 w	458 vw	458 vw	δNC_4_
378 w/369 w	370 vw	373 vw	δNC_4_ τCH_3_
	302 vw-sh	291 vw-sh	
275 s	274 m	261 w	τCH_3_ T′(Cd)
223 vs	220 m	217 w	LN_3_^−^
208 s	186 w	189 w	LN_3_^−^
168 s	161 w	159 w	T’N_3_^−^ T′(Cd)
154 m	149 m	147 m	
134 w	137 w	134 w	
121 m	121 w	117 m	T’(TMA)
116 s/115 m			T’(TMA)
98 w/90 m	101 w/92 vw	101 w/91 w	

Key: vs—very strong, s—strong, m—medium, w—weak, vw—very weak; ν_s_–symmetric stretching, ν_as_—asymmetric stretching, δ_as_—asymmetric bending, δ_s_—δ-symmetric bending (scissoring), ρ—rocking, τ—twisting (torsion), T—translation, L—libration.

## Supplementary Materials

The following are available online, Figure S1: (above) Experimental X-ray powder diffraction pattern of the TMACdN3 sample at room temperature, and (below), the simulated X-ray powder diffraction pattern of TMACdN3 obtained from the single crystal measured data, Figure S2: Perspective view along the c-axis of the crystal structure of the TMACdN3 γ-phase, showing the unconventional cooperative tilting of the [CdN_6_] octahedra, Figure S3: Hirshfeld surfaces of TMACdN_3_ for the α-phase (S.G:C2/c) and γ-phase (S.G.: P21/m) showing in red interactions between the azido ligands in the framework and the TMA cations, Figure S4: Fig. S4. Temperature dependence of the wavenumber of the symmetrical stretch vibrations s(1)N3- and the assignment of azido ligands (right figure). Structural destails of α-phase (S.G: C2/c) and γ-phase (S.G.: P21/m) showing the different crystalloghaphic azido ligands (left image).
