# Supplementary material for: Raman Spectroscopy Studies on the Barocaloric Hybrid Perovskite [(CH3)4N][Cd(N3)3]
Source: Molecules. 2020 Oct 16;25(20):4754. doi: 10.3390/molecules25204754 (PMC7587568; doi:10.3390/molecules25204754)
Supplement: Supplementary file 1 [file molecules-25-04754-s001.pdf]

Raman spectroscopy studies on the barocaloric hybrid perovskite $[(CH_3)_4N][Cd(N_3)_3]$

R. X. Silva[1], C.W.A. Paschoal[2], C.C. Santos[3], Alberto García-Fernández[4], Jorge Salgado-Beceiro[4], María Antonia Señarís-Rodríguez[4], Manuel Sánchez Andújar[4], A. Nonato[5],*

[1]Coordenação de Ciências Naturais, Universidade Federal do Maranhão, Campus VII, 65400-000, Codó-MA, Brazil

[2]Departamento de Física, Universidade Federal do Ceará, Campus do Pici, 65455-900, Fortaleza - CE, Brazil

[3]Departamento de Física, CCET, Universidade Federal do Maranhão, 65085-580, São Luís MA, Brazil

[4]Departamento de Química, Facultad de Ciencias y CICA, Universidade da Coruña, Campus A Coruña, 15071 A Coruña, España

[5]Coordenação de Ciências Naturais, Universidade Federal do Maranhão, Campus do Bacabal, 65700-000, Bacabal - MA, Brazil

corresponding authors: ariel.nonato@ufma.br (A. Nonato) and m.senaris.rodriguez@udc.es (M.A. Señarís-Rodríguez)

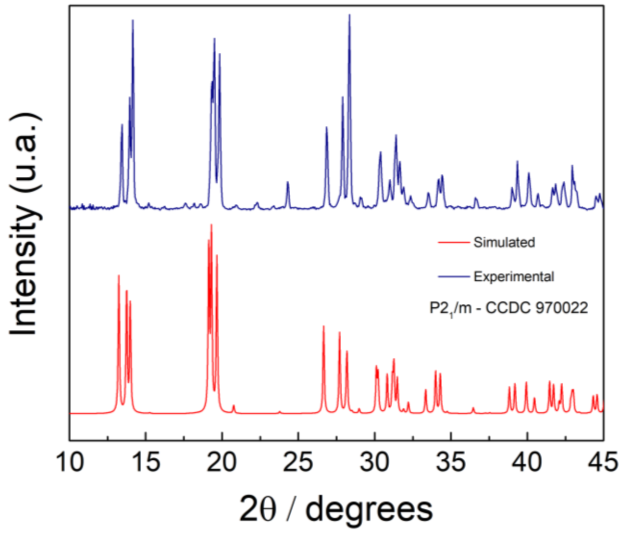

Fig. S1. (above) Experimental X-ray powder diffraction pattern of the TMACdN3 sample at room temperature, and (below), the simulated X-ray powder diffraction pattern of TMACdN3 obtained from the single crystal measured data.

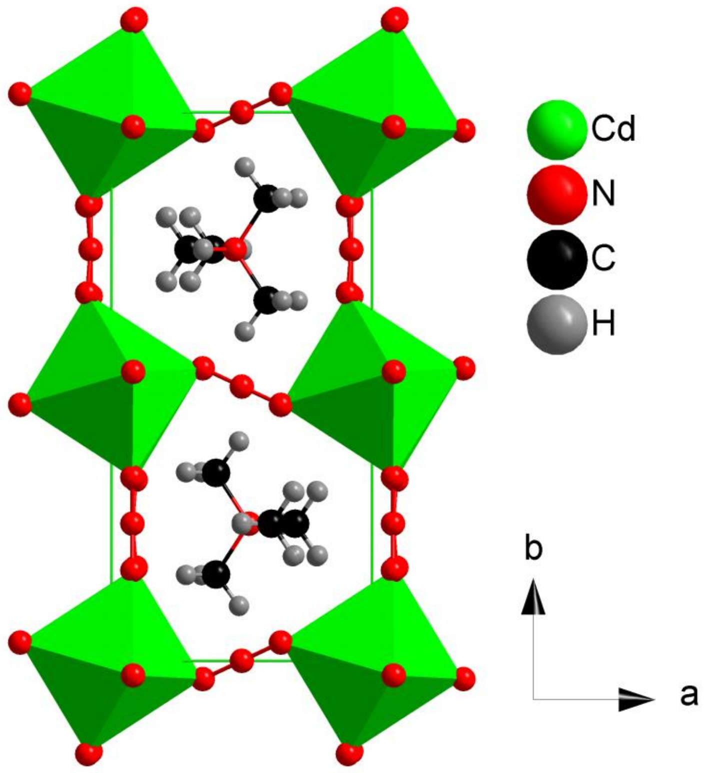

Fig. S2. Perspective view along the c-axis of the crystal structure of the TMACdN3 γ- phase, showing the unconventional cooperative tilting of the [CdN_6] octahedra.

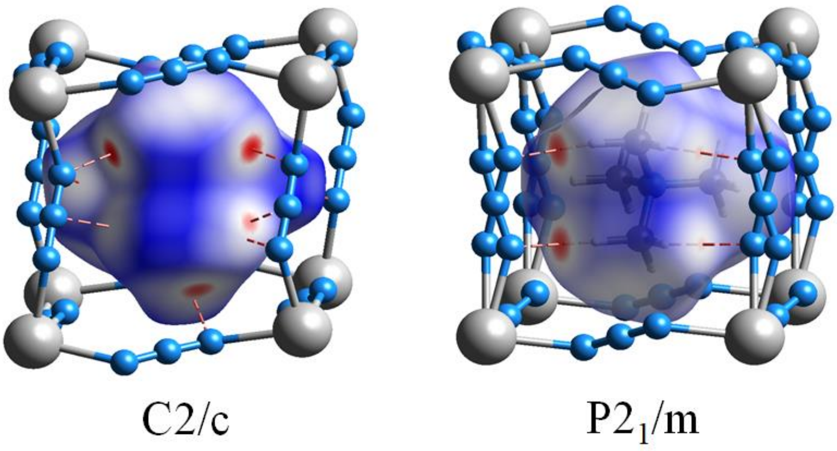

Fig. S3. Hirshfeld surfaces of TMACdN$_3$ for the α-phase (S.G:C2/c) and γ- phase (S.G.: P21/m) showing in red interactions between the azido ligands in the framework and the TMA cations.

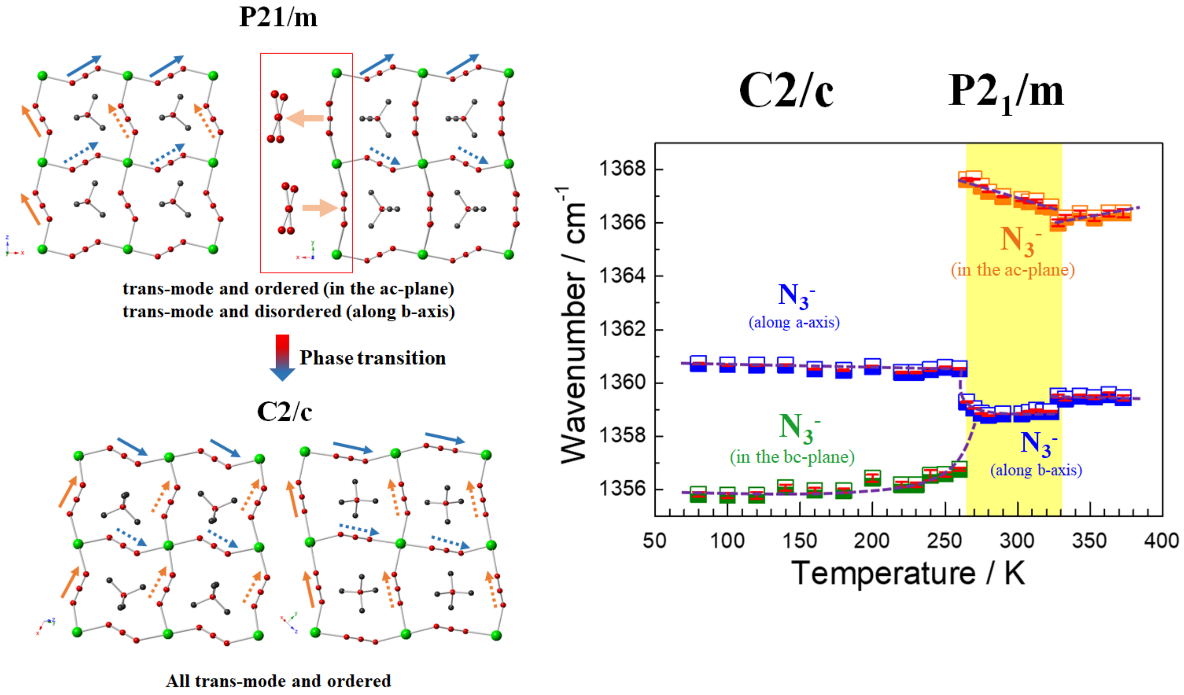

Fig. S4. Temperature dependence of the wavenumber of the symmetrical stretch vibrations $\nu_s(\nu_1)N_3^-$ and the assignment of azido ligands (right figure). Structural destails of α-phase (S.G: C2/c) and γ- phase (S.G.: P21/m) showing the different crystalloghaphic azido ligands (left image).

The factor group analysis

https://www.cryst.ehu.es/rep/sam.html

The space group it has been introduced is $P2_1/m$ (No. 11), its point group is C_{2h} (2/m).

Representation: 38Ag + 43Au + 34Bg + 47Bu (**162**)
Optical modes: $38A_g$+ $42A_u$+ $34B_g$+ $45B_u$ (**159**)
Acoustic modes: A_u+ $2B_u$ (**3**)
Raman-active: $38A_u$+ $34B_u$ (**72**)
IR-active: $42A_u$+ $45B_u$ (**87**)
Silent: –

The space group it has been introduced is $C2/c$ (No. 15), its point group is C_{2h}(2/m).

Representation: $38A_g$+ $41A_u$+ $40B_g$+ $43B_u$ (**162**)
Optical modes: $38A_g$+ $40A_u$+ $40B_g$+ $41B_u$ (**159**)
Acoustic modes: $\Gamma_{acoustic}$= A_u+ $2B_u$ (**3**)
Raman-active: $38A_g$ + $40B_g$ (**78**)
IR-active: $40A_u$+ $41B_u$ (**81**)
Silent: –

Table S1. The factor group analysis for the α phase (*C*2/*c*) of $TMACdN_3$.

Ion	Vibration	Free ion symmetry	Site symmetry	Factor group symmetry
		$D_{\infty h}$	C_1	C_{2h}
(**1**) N_3^-	ν_s (ν_1)	A_{1g}	A	$A_g+A_u+B_g+B_u$
	δ (ν_2)	E_{1u}	2A	$2A_g+2A_u+2B_g+2B_u$
	ν_{as} (ν_3)	A_{1u}	A	$A_g+A_u+B_g+B_u$
	T’	$A_{1u}+E_{1u}$	3A	$3A_g+3A_u+3B_g+3B_u$
	L	$A_{2g}+E_{1g}$[a,*]	2A[a,*]	$2A_g+2A_u+2B_g+2B_u$
		$D_{\infty h}$	C_2	C_{2h}
(**2**) N_3^-	ν_s (ν_1)	A_{1g}	A	A_g+A_u
	δ (ν_2)	E_{1u}	2B	$2B_g+2B_u$
	ν_{as} (ν_3)	A_{1u}	A	A_g+A_u
	T’	$A_{1u}+E_{1u}$	A+2B	$A_g+A_u+2B_g+2B_u$
	L	$A_{2g}+E_{1g}$[a,*]	A+B[a,*]	$A_g+A_u+B_g+B_u$
		T_d	C_2	C_{2h}
(**2**) TMA^+[b]	$\nu_{as}CH_3$	$E+F_1+F_2$	4A+4B	$4A_g+4A_u+4B_g+4B_u$
	ν_sCH_3	A_1+F_2	2A+2B	$2A_g+2A_u+2B_g+2B_u$
	$\delta_{as}CH_3$	$E+F_1+F_2$	4A+4B	$4A_g+4A_u+4B_g+4B_u$
	δ_sCH_3	A_1+F_2	2A+2B	$2A_g+2A_u+2B_g+2B_u$
	ρCH_3	$E+F_1+F_2$	4A+4B	$4A_g+4A_u+4B_g+4B_u$
	τCH_3	A_2+F_1	2A+2B	$2A_g+2A_u+2B_g+2B_u$
	$\nu_{as}NC_4$	F_2	A+2B	$A_g+A_u+2B_g+2B_u$
	ν_sNC_4	A_1	A	A_g+A_u
	δNC_4	$E+F_2$	3A+2B	$3A_g+3A_u+2B_g+2B_u$
	T’	F_2	A+2B	$A_g+A_u+2B_g+2B_u$
	L	F_1	A+2B	$A_g+A_u+2B_g+2B_u$
Cd^{2+}			C_i	C_{2h}
			$3A_u$	$3A_u+3B_u$

red - Raman-active, black -IR-active.

[a]The number of librations is reduced due to the linearity of the azide ion.

[b]A. Mhiri, F. Krichen, A. Oueslati, J. Lhoste, F. Goutenoire, A. Gargouri, A. Bulou, *J. Alloys. Compd.*, 2019, **772**, 546-556.

* – one L is missing (B_g)

** Since the LT phase is based on a centered structure, the number of molecules per unit cell must be reduced by a factor 2. Therefore, for the calculation of the total vibrational modes of the α phase, Z should be considered as equal to 2.
